# Expectant fathers’ knowledge of maternal morbidity: a Sri Lankan experience

**DOI:** 10.12688/f1000research.2-119.v1

**Published:** 2013-05-01

**Authors:** Amaya Weekrakkody, Gihan M Weerasinghe, Mayumi P Weerasinghe, Gayan L Weerasekara, Suneth B Agampodi

**Affiliations:** 1 Department of Community Medicine, Faculty of Medicine and Allied Sciences, Rajarata University of Sri Lanka, Sri Lanka; 2 Faculty of Medicine and Allied Sciences, Rajarata University of Sri Lanka, Mihintale, Sri Lanka

## Abstract

**Background**: Male partners play an important and vital role in the decision-making process regarding pregnant women’s health. The purpose of the present study was to assess the knowledge and awareness of expectant fathers about Gestational Diabetes Mellitus (GDM), Pregnancy Induced Hypertension (PIH), and anaemia during pregnancy.

**Methods**: A cross sectional descriptive study was carried out among expectant fathers whose partners were attending antenatal clinics at the Anuradhapura Teaching Hospital, Sri Lanka. All consenting participants were interviewed by investigators using an interviewer administered questionnaire to collect data on knowledge of risk factors, symptoms, complications and their control. Statistical analysis was performed using the Kruskal Wallis test.

**Results**: Of the 246 expectant fathers studied, 192 (78%) were aware of GDM, 183 (74.4%) and 154 (62.6%) were aware of PIH and anaemia during pregnancy, respectively. The total number of answers provided by expectant fathers ranged from 0 to 33 (of 41 questions). There were 44 fathers who could not answer even a single question. For GDM, anaemia, and PIH, the percentages of expectant fathers who failed to provide at least a single correct answer were 24.8%, 40.2%, and 31.3%, respectively. The median number of total correct answers provided increased steadily along with the average income (chi-square 31.24, p<0.001) and educational level (chi-square 33.57, p<0.001). Expectant fathers in the 25-34 age group had significantly higher scores, compared to younger and older fathers (chi-square 15.11, p=0.001). Fathers experiencing the second pregnancy of their spouses also had higher scores.

**Conclusions**: Expectant father’s knowledge of the selected morbidities was limited. To improve maternal health, any health promotional programmes should include expectant fathers.

## Introduction

The important role of men as the primary stakeholders of women’s reproductive and sexual health has been widely accepted. In 1994, 20,000 United Nations delegates with member status pressed for men’s involvement in reproductive health. The programme of action of the International Conference on Population and Development (ICPD) stressed men’s shared responsibility for women’s health ^1^. Although the primary focus was on reducing violence against women and children, the resolution clearly declared that men should take responsibility for the empowerment of women.

Studies on family planning ^2, 3^, HIV/AIDS ^4^, abortions ^4^ and breastfeeding ^5^ all provide clear evidence for the important and vital role of male partners in the decision-making process regarding women’s health. Interventional studies on reproductive health programmes targeting either males or both sexes, compared to programmes targeting only females, have shown a significant effect on the reproductive health outcome of women. With this growing body of evidence, the concept of men as “gatekeepers” of women’s health has been developed into a positive approach that includes men as partners in improving women’s health.

Nevertheless, these involvements are often driven by the gender power relationships given to male partners in various cultures ^6^. While reproductive health education and health promotion programmes that target adolescent boys and young men are acceptable in most parts of the world, shared responsibility for pregnancy-related matters heavily depends on the nature of gender roles within the society. In addition, fear of losing respect from peers, lack of communication skills, lack of knowledge, and perceptions of masculinity are also shown to have a major effect on this involvement. Despite having a lack of knowledge and involvement, men often dominate the decision-making processes related to pregnancy, especially in male-dominated South Asian cultures.

Studies in maternal health have shown that early interventions during the antenatal period have a major protective effect for medical conditions complicating pregnancy and direct pregnancy-related acute complications such as haemorrhage, rupture of the uterus, and obstructed labour. These interventions include early identification, early treatment and behaviour modifications.

However, screening tests and procedures for these conditions require resources, and early detection is not currently at an optimal level in most developing countries. We hypothesized that this could be partly due to a lack of awareness about maternal morbidity among pregnant women, and specifically among their partners who play a major role in decision-making. The knowledge of expectant fathers is vital to ensure that healthcare is sought early to prevent complications due to these conditions. The purpose of the present study was to assess the knowledge and awareness of expectant fathers about Gestational Diabetes Mellitus (GDM), Pregnancy Induced Hypertension (PIH), and anaemia during pregnancy.

## Methods

This study conformed to the Helsinki Declaration and to local legislation. All participants gave informed consent to participate in this study. Ethical clearance was obtained from the Research and Ethics Committee, Faulty of Medicine and Allied Sciences, Rajarata University of Sri Lanka.

The study was conducted in Anuradhapura, Sri Lanka, during November to December 2010. Anuradhapura district is situated in the North Central Province of Sri Lanka. The resident population is around 830,000 ^7^. Over the five years preceding this study the annual number of births reported from the district was around 16,000. The crude birth rate is 20.7/1000 population. Infant and neonatal mortality rates of Anuradhapura district (17.2/1000 live births (LB) and 11/1000 LB) are significantly higher than those reported in Sri Lankan national data, which are 11/1000 LB and 8.7/1000 LB, respectively ^8^.

The study population for the present study included expectant fathers whose partners were attending antenatal clinics at the Anuradhapura Teaching Hospital. The study sample included the expectant fathers that were accompanying pregnant women to the antenatal clinics. The participants included fathers who were waiting during the clinic hours as well as the fathers who came to the clinic just to drop off the pregnant women at the clinic (this was a common practice and usually they returned around four hours later). The authors visited the antenatal clinics at the starting time and introduced themselves to expectant fathers, explained the objectives of the study, and handed out the self-administered questionnaires. The study sample for the present study included all expectant fathers visiting selected clinics during the study period. Since the study followed a non-probability sampling procedure, the optimal sample size was not calculated. However, it was hypothesized that if the 80% of the expectant fathers are aware of the selected morbidities and a simple random sampling procedure was followed, a minimum of 246 expectant fathers were required with 95% confidence limits and 5% absolute precision.

Variables for the study included basic socio-demographic variables and knowledge about GDM, PIH and anaemia. For each condition selected, expectant fathers were first asked about their awareness of the existence of certain disease conditions, for example, ‘Are you aware that pregnant women could develop a condition called gestational diabetes mellitus?’. Then a selected set of questions on risk factors, clinical features, complications and preventative measures (primary and secondary) was included for all selected conditions (e.g., ‘Which of the following could be risk factors for developing GDM?’). Only true risk factors, features and preventative measures were provided in the questionnaire. This approach was followed as a way of including health education and to avoid the possibility of giving incorrect messages through the survey. The variables were selected after discussion with medical, obstetric, and community medicine experts. The questionnaire was developed in English and translated into Sinhalese.

All demographic variables were analyzed as categorical variables. Answers to individual questions were presented as percentages. The total number of correct answers provided by participants was also reported as a percentage. The knowledge distribution was expected to have a skewed distribution because of the distribution of educational status in this population is also skewed, and non-parametric tests were carried out for significance testing.

## Results

The total number of expectant fathers studied was 246. The mean age of the participants was 30.2 years (standard deviation (SD) of 6.2 years) and the mean age of the pregnant mothers was 26.6 years (SD of 5.6 years). Table 1 shows the demographic characteristics of the study sample.

**Table 1.** Characteristics of 246 expectant fathers who participated in the Knowledge Assessment Survey on GDM, PIH and anaemia.

Characteristic	n	(%)
Highest level of education		
Never been to school	7	2.8
Grade 1–5	14	5.7
Grade 6–10	53	21.5
GCE O/L	115	46.7
GCE A/L	50	20.3
Diploma/degree	7	2.8
Ethnicity		
Sinhalese	226	91.9
Tamils	5	2.0
Moor/Malay	15	6.1
Average monthly income (SLR)		
<5000	23	9.3
5001–15,000	134	54.5
>15,000	89	36.2
Parity of partner		
1	117	47.6
2	81	32.9
3	31	12.6
4	14	5.7
5	1	0.4
6	2	0.8

GCE O/L-General certificate in Education Ordinary Level.

GCE A/L-General certificate in Education Advanced Level.

The prevalence of GDM, PIH and anaemia during the present or previous pregnancies as reported by the fathers was 6.9% (n=17), 4.5% (n=11) and 11.4% (n=28), respectively. Of the 246 expectant fathers studied, 192 (78%) were aware of GDM, and 183 (74.4%) and 154 (62.6%) were aware of PIH and anaemia during pregnancy, respectively.

Of the risk factors listed in the questionnaire, a family history of GDM/diabetes mellitus (DM) was recognized by 48.8% of the study sample ( Table 2). Of the respondents, 135 (54.9%) knew that increased frequency of urination was a common clinical presentation for GDM/DM. The probability of developing noninsulin-dependent diabetes mellitus (NIDDM) in later life as a complication of GDM was known to 98 (39.8%) respondents. Screening for GDM if risk factors were present was indicated as an appropriate intervention to control GDM by 30.5% (n=75) of participants.

**Table 2.** Percentage of expectant fathers who identified probable risk factors, clinical features, complications and control/preventive measures of GDM, PIH and anaemia.

	n	%
**GDM Risk factors**		
Family history of DM	120	48.8
Age > 35 years	66	26.8
GDM in previous pregnancies	62	25.2
**GDM Clinical features**		
Increased frequency of urination	135	54.9
Abnormal increase of weight during gestational period	35	14.2
Polyphagia	46	18.7
Recurrent urinary tract infections	12	4.9
**GDM Complications**		
Diabetes in later in life	98	98
Delivery of an abnormally large baby	17	6.9
Diabetes in child	51	20.7
Neonatal problems	6	2.4
Obstructed labor	11	4.5
**GDM Control and prevention**		
Perform a blood glucose test in the booking visit/ early in pregnancy	71	28.9
Perform specific blood tests if risk factors/clinical symptoms present	75	30.5
**Anaemia Risk factors**		
Pregnant mothers age > 35 years	66	26.8
Increased parity (more than 5 children)	11	4.5
Poor spacing (less than 2 years)	11	4.5
**Anaemia Clinical features**		
Lethargy	77	31.3
Severe headache	92	37.4
Pale lips/tongue	87	35.4
**Anaemia Complications**		
Stillbirths	36	14.6
Restriction of intra uterine growth	17	6.9
Low birth weight	14	5.7
Postpartum hemorrhage	65	26.4
**Anaemia Prevention**		
Adequate intake of iron rich food	123	50.0
Avoid taking tea, coffee or milk with meals	45	18.3
Add lime juice during preparation of green leaves	22	8.9
**PIH Risk factors**		
Age more than 40 years	53	21.5
Chronic hypertension	55	22.4
Chronic renal failure	3	1.2
**PIH Clinical features**		
Dizziness	143	58.1
Edema in heel or dorsal aspect of foot	51	20.7
High blood pressure	38	15.4
Double vision	20	8.1
**PIH Complications**		
Stillbirths	33	13.4
Bleeding during antenatal period	12	4.9
Pre-term delivery	19	7.7
Low birth weight	30	12.2
**PIH Control and prevention**		
Bed rest	18	7.3
Check blood pressure in every visit	105	42.7
Take proper medications for high blood pressure	115	46.7

Increasing maternal age was the risk factor most commonly recognized among expectant fathers as a risk factor for developing anaemia during pregnancy, which was reported by 26.8% (n=66) of the fathers. All other risk factors were known to less than 15% of the respondents. However 92 fathers (37.4%) reported headache as a common clinical presentation of anaemia, and 65 (26.4%) were aware of the increased risk of postpartum haemorrhage. Half of the study sample responded that adding iron-rich food to the diet would help to prevent anaemia. Dizziness was identified as a probable symptom of PIH by 58.1% (n=143) participants. The most well known complication of PIH was stillbirth (13.4%). Nearly half of the participants agreed that PIH needed pharmaceutical intervention for proper control.

The total number of answers provided by the expectant fathers ranged from zero to 33 (of 41 questions). The distribution of the total scores showed a highly-skewed distribution with majority aggregating towards low scores. There were 44 fathers who could not answer even a single question. For GDM, anaemia, and PIH, the percentage of expectant fathers who failed to provide at least a single correct answer was 24.8%, 40.2%, and 31.3%, respectively.

**Figure 1.**
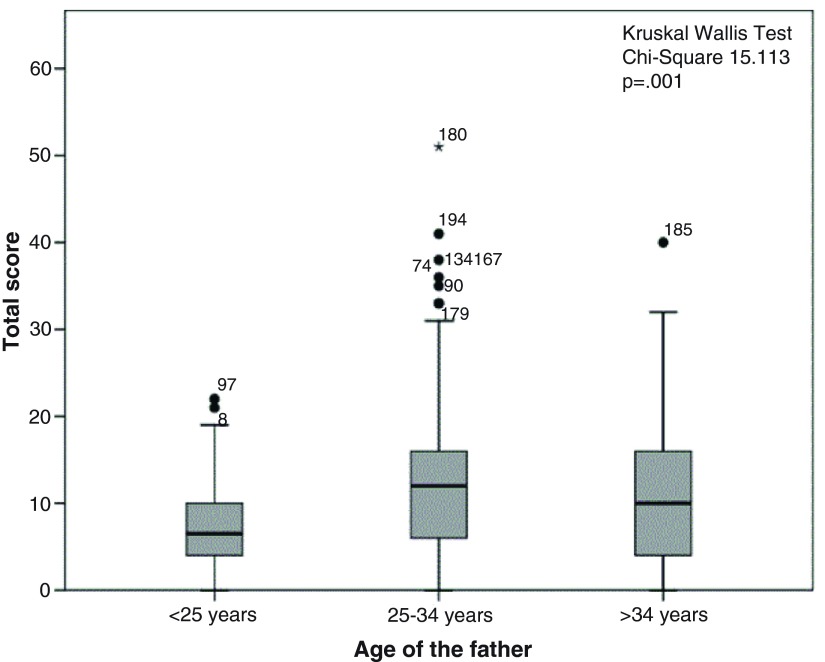
Distribution of total score by age among 246 expectant fathers who participated in the Knowledge Assessment Survey on GDM, PIH and Anaemia. (The box covers the Interquartile range (IQR); whiskers extend 1.5 IQR from the box; circles shows outliers lying between 1.5 to 3 IQR from box and the asterisk shows extremes more than 3 IQR from box. Numbers denotes the identification number for the study unit).

The distribution of score totals by socio-demographic characteristics is illustrated in Figure 1– Figure 5. Median scores increased steadily along with the average income and educational level. Expectant fathers in the 25–34 age group had significantly higher scores, compared to younger and older fathers. Fathers experiencing the second pregnancy of their spouses also had higher scores. Ethnic group was not associated with the scores obtained in this study sample.

**Figure 2.**
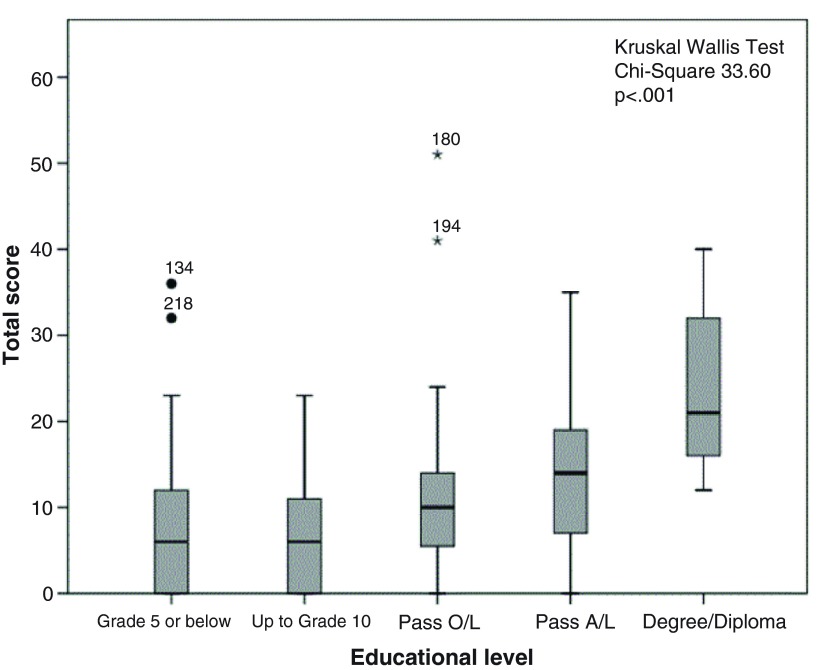
Distribution of total score by educational attainment among 246 expectant fathers who participated in the Knowledge Assessment Survey on GDM, PIH and Anaemia. (The box covers the Interquartile range (IQR); whiskers extend 1.5 IQR from the box; circles shows outliers lying between 1.5 to 3 IQR from box and the asterisk shows extremes more than 3 IQR from box. Numbers denotes the identification number for the study unit).

**Figure 3.**
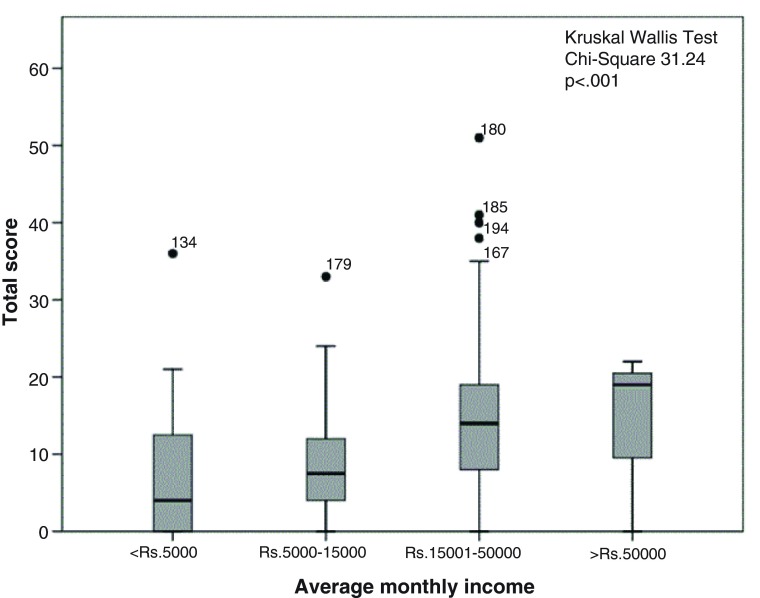
Distribution of total score by average monthly income among 246 expectant fathers who participated in the Knowledge Assessment Survey on GDM, PIH and Anaemia. (The box covers the Interquartile range (IQR); whiskers extend 1.5 IQR from the box; circles shows outliers lying between 1.5 to 3 IQR from box and the asterisk shows extremes more than 3 IQR from box. Numbers denotes the identification number for the study unit).

**Figure 4.**
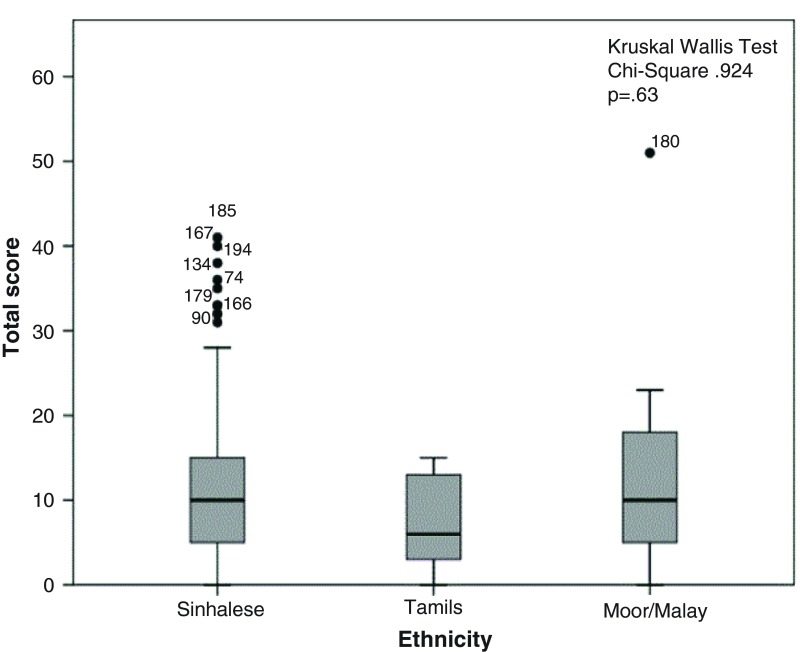
Distribution of total score by ethnicity among 246 expectant fathers who participated in the Knowledge Assessment Survey on GDM, PIH and Anaemia. (The box covers the Interquartile range (IQR); whiskers extend 1.5 IQR from the box; circles shows outliers lying between 1.5 to 3 IQR from box and the asterisk shows extremes more than 3 IQR from box. Numbers denotes the identification number for the study unit).

**Figure 5.**
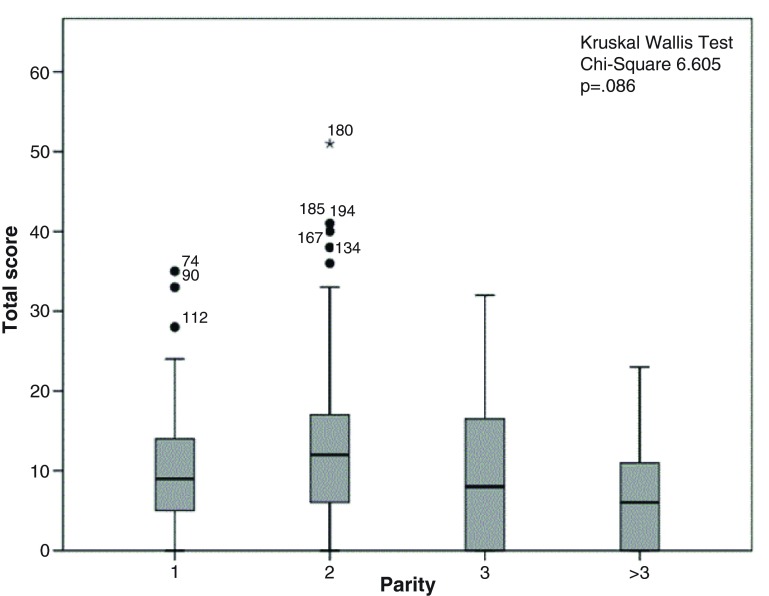
Distribution of total score by parity of the spouse among 246 expectant fathers who participated in the Knowledge Assessment Survey on GDM, PIH and Anaemia. (The box covers the Interquartile range (IQR); whiskers extend 1.5 IQR from the box; circles shows outliers lying between 1.5 to 3 IQR from box and the asterisk shows extremes more than 3 IQR from box. Numbers denotes the identification number for the study unit).

Paternal knowledge of maternal morbidity in Sri Lanka Collated answers to questionnaire delivered to expectant fathers.

## Discussion

Pregnancy and childbirth have long been considered as a women’s domain ^9^ by communities in which the involvement of male partners is minimal. Some studies even suggest that the number of expectant fathers seeking healthcare for their spouse’s problems is lower than that of non-expectant fathers ^10^. The reason for this could be due to the fact that women are more likely to persuade men to access healthcare rather than men being able to persuade women to seek help ^11^. However, the intentions of both partners have shown to be a better predictor of health behavior ^12^; thus the father’s knowledge is vital in health-seeking behavior during pregnancy. The present study provides evidence to show that the expectant fathers have a limited knowledge of the main maternal morbidities. As this study showed that there was very poor knowledge, the results could not be interpreted, but there is definitely room for improvement.

Anemia was assumed to be a well-known condition among Sri Lankan people due to several factors. It is a major determinant in maternal health, and the public health programme in Sri Lanka has conducted endless campaigns to prevent this condition over the last few decades. In a study done in 2009–10 period, the prevalence of anemia among pregnant women in Anuradhapura was reported as 14% ^13^. However, the present study shows that in Anuradhapura, 37.4% of fathers were not even aware that anemia could be a problem during pregnancy. This raises a major concern regarding the effectiveness of the present health education programmes. Either these programmes are only directed at pregnant mothers, or the programmes have failed to convey their essential messages.

Knowledge of GDM was slightly better than for other conditions, most likely due to the increasing community prevalence of diabetes in Sri Lanka, because the knowledge of the direct complications in pregnancy associated with GDM was poor. Prevalence of GDM in Sri Lanka is increasing, and a community-based study showed that around 10.3% of pregnant mothers in Sri Lanka experience GDM ^14^. The present screening programme for GDM is not functioning well, due to a lack of facilities. If the community, especially expectant fathers, can be made aware of this condition, it will change care-seeking behavior and more cases will be detected and treated, thus preventing severe complications to both mother and child.

Eclampsia and PIH are listed as the second leading cause of maternal deaths in Sri Lanka. The condition accounts for a considerable proportion of hospitalizations, and the disease burden is very high. Routine blood pressure and urine albumin measurements are carried out in antenatal clinics as screening tests for PIH. In this study sample, risk factors and complications of PIH were known to a limited number of expectant fathers. However, dizziness, which is a non-specific sign of PIH, was known to more than 50% of the study sample as a clinical manifestation.

One major observation related to all three conditions was lack of knowledge about the complications of selected conditions. This lack of knowledge reduces the perception of risk due to these conditions. Risk perception is a core concept of health behavior, which is largely based on health literacy ^15^. The study results suggest that the Sri Lankan maternal health programme should change their strategies in order to improve knowledge about these conditions.

Exploratory models have shown that health risk perception which is the basis for care-seeking depends on social class ^16^, socioeconomic determinants, and the structure and practices of the health system ^17^. Among pregnant mothers, knowledge about pregnancy risk factors ^18^ and GDM has been shown to be associated with educational status ^19^. We also observed that level of education and income are positively associated with expectant fathers’ knowledge about maternal morbidities.

Such inequalities in social factors seriously affect heath and underpin all other determinants of health. Sri Lanka has shown remarkable success in overcoming these social inequalities in reducing maternal deaths. However, the maternal morbidity pattern still reveals a high level of inequality, and poor knowledge among expectant fathers could be one major determinant of the problem.

## Limitations

The study sample consisted of expectant fathers who were visiting clinics. These fathers may be more health conscious and not representative of the total population of expectant fathers. The knowledge observed may be higher than the actual prevailing knowledge. The majority of the individuals in the study sample were visiting antenatal clinics at the teaching hospital in Anuradhapura. These fathers might represent a group of fathers whose socioeconomic background was different from fathers living in rural areas. Some of the questions included in the questionnaire were testing a very high level of health literacy. However, the percentage of correct answers was only used as a subjective score in the interpretation.

## Conclusions

The limited level of knowledge among expectant fathers clearly shows room for the improvement of maternal health programmes. We suggest that safe motherhood programmes should have practical and operational strategies to include expectant fathers in maternal health promotion programmes.

## Consent

All participants gave informed consent to participate in this study. Ethical clearance was obtained from the Research and Ethics Committee, Faulty of Medicine and Allied Sciences, Rajarata University of Sri Lanka.

## Figures and Tables

**Appendix 1:**
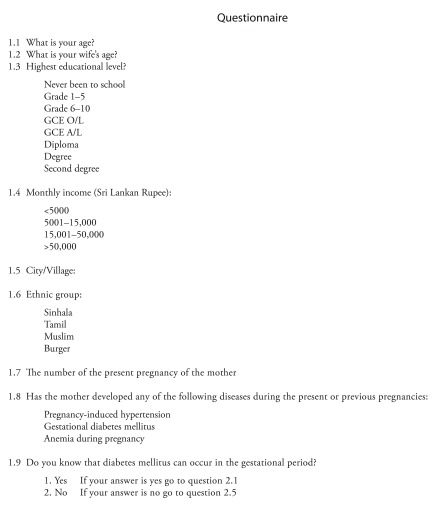
Questionnaire of paternal knowledge of maternal morbidity in Sri Lanka (English). (Sheet 1).

**Appendix 1:**
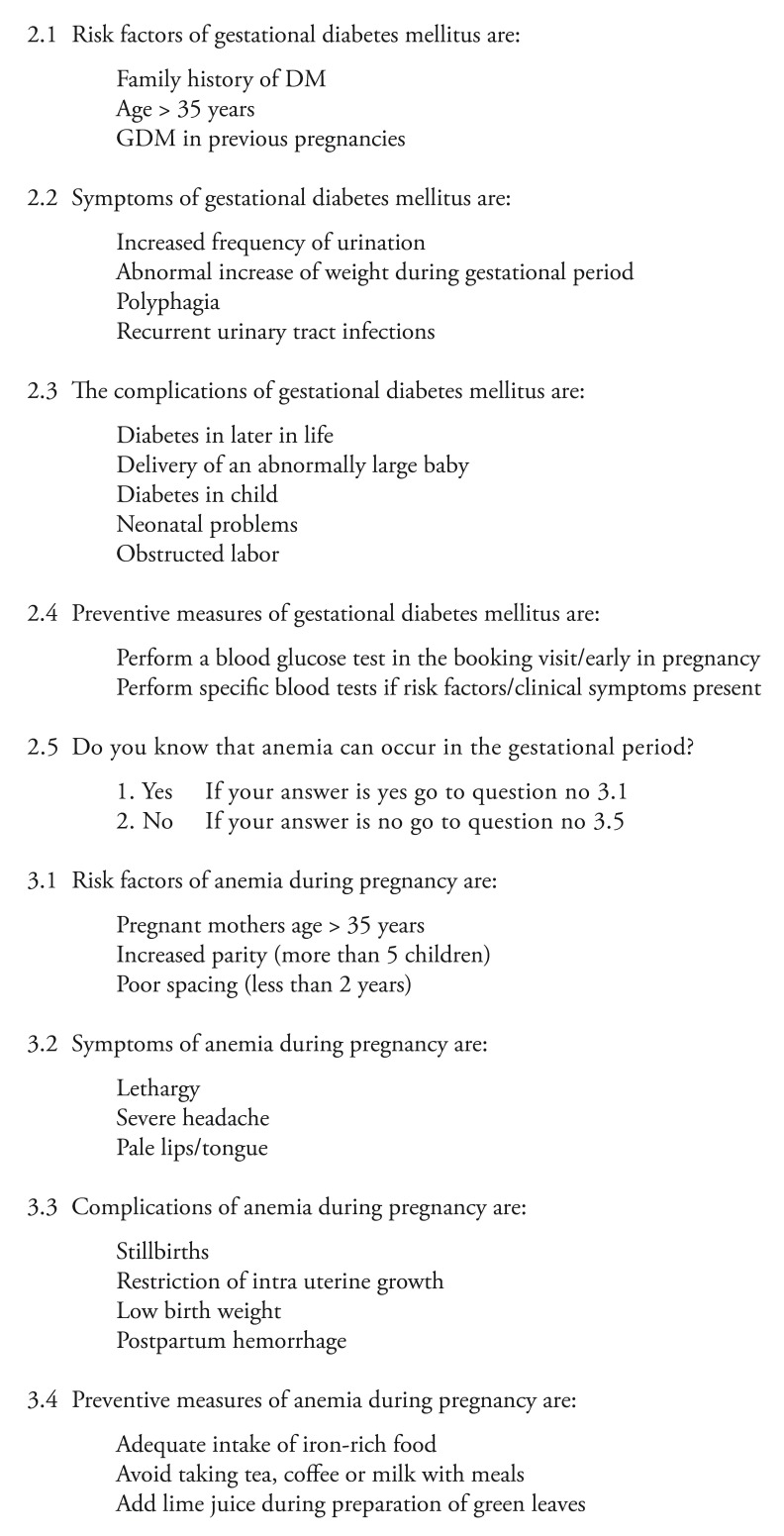
Questionnaire of paternal knowledge of maternal morbidity in Sri Lanka (English). (Sheet 2).

**Appendix 1:**
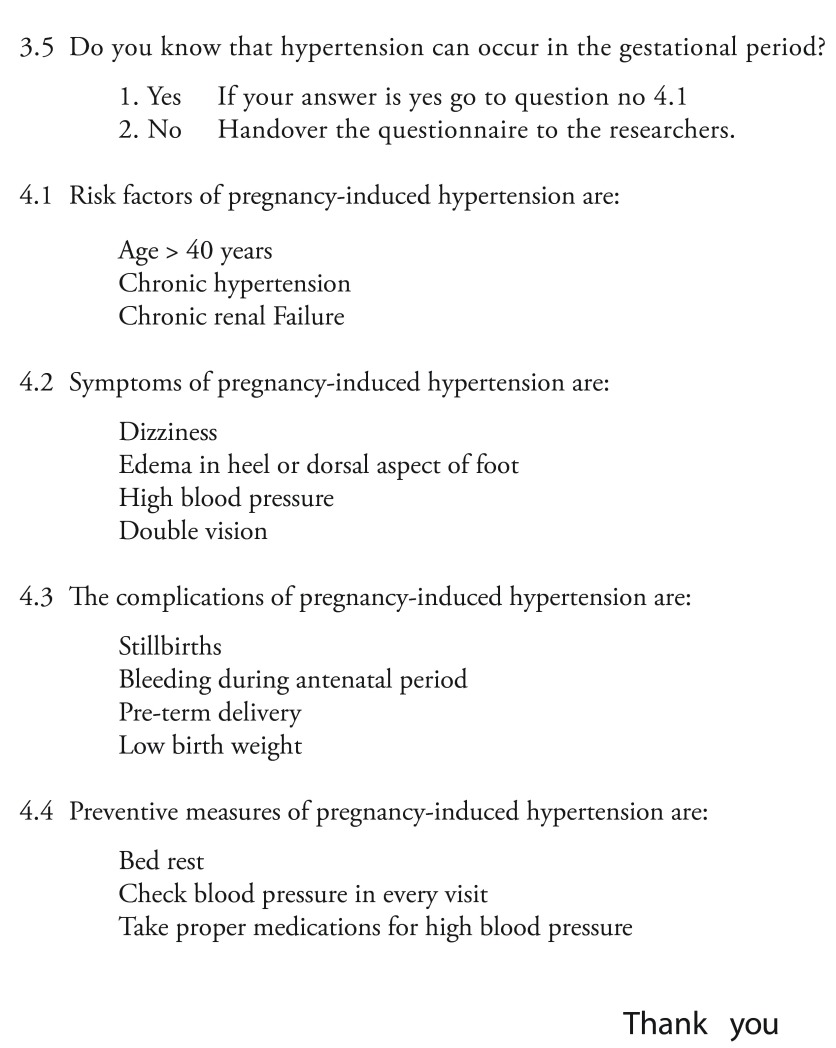
Questionnaire of paternal knowledge of maternal morbidity in Sri Lanka (English). (Sheet 3).

**Appendix 1:**
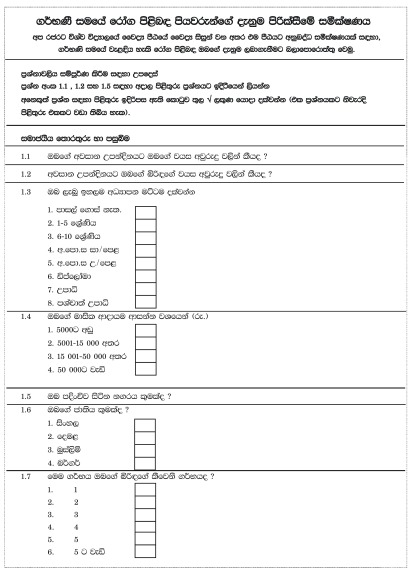
Questionnaire of paternal knowledge of maternal morbidity in Sri Lanka (Sinhalese). (Sheet 1).

**Appendix 1:**
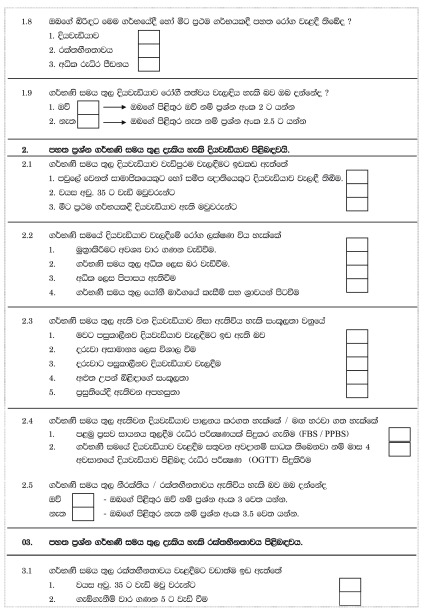
Questionnaire of paternal knowledge of maternal morbidity in Sri Lanka (Sinhalese). (Sheet 2).

**Appendix 1:**
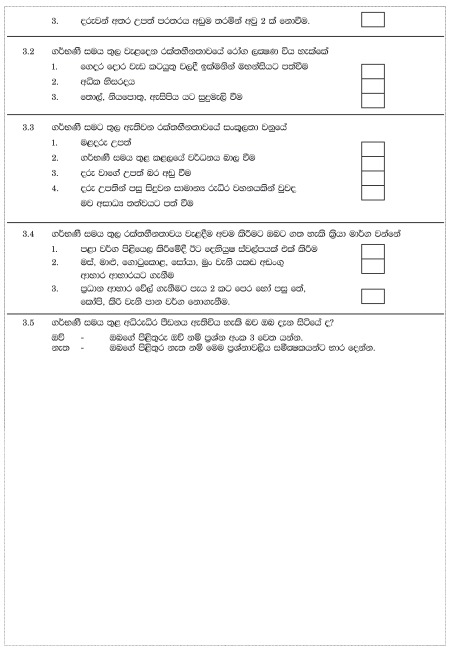
Questionnaire of paternal knowledge of maternal morbidity in Sri Lanka (Sinhalese). (Sheet 3).

**Appendix 1:**
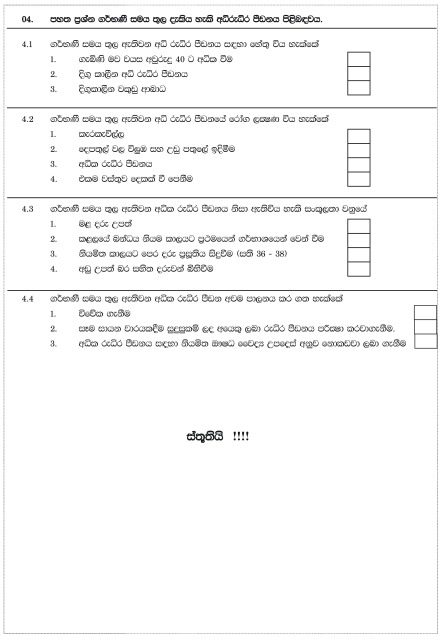
Questionnaire of paternal knowledge of maternal morbidity in Sri Lanka (Sinhalese). (Sheet 4).
