# Supplementary material for: Expectant fathers’ knowledge of maternal morbidity: a Sri Lankan experience
Source: F1000Res. 2013 May 1;2:119. [Version 1] doi: 10.12688/f1000research.2-119.v1 (PMC4184315; doi:10.12688/f1000research.2-119.v1)
Supplement: Peer review history

# Peer review: Referee response for version 1

**DOI:** 10.5256/f1000research.1279.r4139

**Published:** 2014-03-17

**Authors:** Shahirose Premji

**Affiliations:** 1 Faculty of Nursing, University of Calgary, Calgary, Canada

I wish to extend my appreciation for the invitation to referee this important study which examines expectant fathers’ knowledge and awareness about pregnancy complications – gestational diabetes mellitus, pregnancy-induced hypertension, and anaemia – that may impact health outcomes of both the mother and baby. The relevance of the work undertaken is well articulated in the introduction and the authors used a non-experimental descriptive research design to assess expectant fathers’ knowledge and awareness about selected morbidities. Fathers were recruited from antenatal clinics at the Anuradhapura Teaching Hospital. Eighteen percent (44 of 246) of expectant fathers were unable to answer a single question (awareness and knowledge). The remaining fathers (82%) demonstrated varied levels of awareness about gestational diabetes mellitus (78%), pregnancy-induced hypertension (74%), and anaemia (63%) and knowledge regarding risk factors, signs and symptoms, associated complications, and management strategies. The findings have implications for Sri Lankan maternal health programme intent to improve health literacy of the population and strategies to promote health seeking behaviors.    

**Major Comments**

**Introduction:**

- **Hypothesis: **The statement leading to the hypothesis emphasizes health inequities (i.e. lack of resources related to screening and managing morbidities); however this is not the focus of the study. Moreover, the hypothesis would suggest that the authors wish to explain why some expectant mothers and fathers seek care while other don’t; asserting that lack of knowledge and awareness may be one factor. The way in which the hypothesis is stated would suggest that the study examines both expectant mothers’ and expectant fathers’ knowledge and awareness about selected morbidities. The descriptive nature of the study would suggest that you want to determine whether expectant fathers whose partners are seeking care have increased knowledge and awareness given their role in decision making about seeking care.

**Design and Methods:**

- **Setting: **Provide a more detailed description of the study setting to determine how representative the sample is of the population seeking care at this hospital. For example, whether it is common practice for expectant fathers to accompany their partner (i.e. wait with then or drop them off) to the antenatal clinic, and the average number of women who attend the clinic per day, to give perspective for the short duration of recruitment (November to December 2010).
- **Recruitment: **How did you enroll the expectant fathers who dropped off their partners at the clinic? Your demographic description should indicate the numbers of expectant fathers who were waiting with their partners and the number who were dropping their partner off. Provide more detail related to the recruitment process (e.g. the number who refused to participate, language use, if unable to sign how was this managed, what were potential risks/harms and how did you manage these?).
- **Questionnaire: **Provide more details. The English version of the questionnaire uses medical terms (e.g., gestational diabetes mellitus, Polyphagia) which participants may not be familiar with. For example, in some cultures diabetes is referred to as sugar in the blood. When translating the English to Sinhalese, was the language appropriately changed? What was the level of readability in the Sinhalese language? Was the questionnaire back-translated to English to ensure language equivalency? Was the questionnaire piloted? Given the variability in literacy (i.e., levels of education) within the community, how did participants complete the questionnaire? For instance, did the researcher or research assistant assist by using an interview format? On page 3 it is stated that “The total number of answers provided by the expectant fathers ranged from zero to 33 (of 41 questions)”. The questionnaire shared in Appendix 1 only has 23 questions. Explain how each of the questions, particularly those with multiple components (e.g., risk factors) was presented and how responses were elicited.  

 

**Analysis:**

- You indicate “All demographic variables were analysed as categorical variables”; however report the mean age of participants and their partners. Were data normally distributed to report means? Revise this statement accordingly. 

**Results:**

- First paragraph: provide a brief description of your sample by highlighted distinct characteristics. For instance, for most women this was their first pregnancy. In the discussion comment on how your sample compares to the general population (i.e., how representative is it). Is it mostly first time mothers who access antenatal clinics?
- Did all expectant fathers provide demographic information? If so, include n of the sample in the table.
- You provide data on the prevalence of gestational diabetes mellitus, pregnancy induced hypertension, and anaemia as reported by fathers (page 3 second paragraph of results). There are two issues with this statement: (1) prevalence refers to the proportion of a population. Your study used a convenience sample. (2) The estimate was based on fathers’ report but many of the fathers were not able to recognize these morbidities. Please clarify.
- Findings: need to be presented more clearly.
   
  - The missing data needs to be accounted for in the tables and explanations provided in the text.
  - Please explain how the percentages were calculated. There are instances when the total percent count is over 100% (e.g. GDM risk factors) and in some instance well below 100% (e.g. GDM complications).
  - Did all participants answer questions related to demographic characteristics? If so, an important consideration would be describing how similar or different the demographic characteristics were between respondents and non-respondents.
  - Examination of the distribution of responses based on specific demographic variables: Explain what you mean by “score totals”m and how this score was determined? Given the multiple comparisons being made, you are likely to find a significant difference by chance (i.e., Type 1 error).  Consider the p value adjustments required for these multiple comparisons. These findings should be interpreted with caution given the nature of the questionnaire (i.e., medical terms used), the type of design, and number of participants who were able to respond. 

 

**Discussion:**

- The first paragraph discusses expectant fathers seeking health care and non-expectant fathers seeking health care, which is not the focus of your study. Delete.
- Needs more depth and breadth by addressing:
   
  - Findings regarding awareness: was this what you expected given the policy changes (i.e., campaigns in the country) over the last 5 years? Why may the findings not be in keeping with what you expected?
  - Lack of knowledge may reduce risk appraisal which guides health behaviour (i.e. seeking care); hence how do you explain the health care seeking behaviour of these couples? What else may be guiding their health seeking behaviors? The World Health Organization recommends 4 antenatal clinic visits – what is the potential implication of the finding of your study with regarding to compliance to this recommendation.
  - Knowledge about selected morbidities: discuss every aspect evaluated (e.g. risk factors, signs and symptoms, etc.). What can be gleaned from these findings? Are similarities and differences? Would you expect the population have knowledge about management?
  - Discuss the role of parity with reference to health seeking behaviors and knowledge or health literacy.
  - Be careful with the inferences you are making. For example, improving knowledge will improve health seeking behavior. You findings suggest that expectant fathers seek health care for their pregnant spouse even though they don’t have the knowledge.
  - Refocus the discussion with emphasis on the findings and implications for practice, future research and policy. 

**Limitations:**

1. Consider revising after you have addressed the above points. For instance, if medical terms were used, this may explain why so many respondents were not able to complete even one question.

 

**Quality of written English:**

- Results and Discussion: a few sentences which are not clear. Page 3, last paragraph has a couple of sentences which are not clear. Page 7, discussion, first paragraph, second sentence and last sentence in the paragraph. Page 7, last paragraph beginning knowledge of GDM delete “because the knowledge of the direct complications in pregnancy associated with GDM was poor” as this does not explain the finding. Was there another point you were trying to make? Please clarify. Page 8, limitations, last sentence in the paragraph – not sure what you mean. 

 

**Minor Comments:**

- **Title:** insert “selected” after knowledge of.
- **Abstract:** The results section may need to be modified after you have addressed the methodological issues.
- **Language: **change developing country to lower middle income (The World Bank income level categorization for Sri Lanka).
- **Tables and Figures: **Ensure all abbreviations are written out in full at the bottom.
- **Figures 1-5: **present in the order in which they are discussed in the text.  

I have read this submission. I believe that I have an appropriate level of expertise to confirm that it is of an acceptable scientific standard, however I have significant reservations, as outlined above.

# Peer review: Referee response for version 1

**DOI:** 10.5256/f1000research.1279.r1119

**Published:** 2013-07-18

**Authors:** Tabassum Firoz

**Affiliations:** 1 University of British Columbia, Vancouver, BC, Canada

This is an important body of work as men's participation is critical in achieving good maternal health outcomes.

I would caution the authors from making a statement that men are the 'primary stakeholders' or 'gatekeepers' in women's health. While you make the point that is certainly true in many societies, it important to keep in mind that how women access care is more complex. The root causes which are economic and social go beyond men.

A minor point about semantics: I would avoid using PIH- either use gestational hypertension (or pre-eclampsia- I could not tell from the questions what you were targeting as these two entities are very different while being on the same spectrum). Secondly, instead of 'developing countries', I would suggest 'low income countries'.

I am concerned that the language and the level of knowledge required to understand the questionnaire is indeed very high. You do rightfully point this out. Can you explain why you chose such a sophisticated level? How does this relate back to your objective of assessing knowledge around morbidity? The questions are at the level a health care professional might answer. A small percentage of your sample didn't even have any education! Most questionnaires should be targeted to a grade 6 (by North American standards) level.

From a public health perspective, the key messages should be to recognize that a woman is sick (perhaps even have a certain condition in mind) and to know what actions should be taken to prevent and/or treat the condition. Most certainly, the key is to seek care as soon as illness is recognized. I don't think the questions reflect this.

Secondly, some of the choices are wrong- for example, anemia does not present with severe headache. In fact, many serious conditions in pregnancy present with headache and this gives the impression that a less severe condition like anemia can present like this. Heel edema/ankle edema may be present in normal pregnant women and therefore, it is not a good choice. Bed rest is actually not recommended for the hypertensive disorders of pregnancy.

It would be interesting to know how men whose partners previously had these conditions scored compared to men whose partners did not have these conditions.

I think the authors need to revise the discussion section with more limitations and as well propose how this knowledge can be used.

I have read this submission. I believe that I have an appropriate level of expertise to confirm that it is of an acceptable scientific standard, however I have significant reservations, as outlined above.

# Peer review: Referee response for version 1

**DOI:** 10.5256/f1000research.1279.r1033

**Published:** 2013-07-01

**Authors:** Laxmi Baxi

**Affiliations:** 1 Department of Obstetrics and Gynecology, Columbia University Medical Center, New York, NY, USA

This is an interesting manuscript, which addresses the awareness of expectant fathers about health issues in expectant mothers. Clearly, health issues in pre-pregnant state and during pregnancy contribute towards compromised maternal and neonatal-infant health including mortality and such information is not commonly addressed in the literature. The authors have mentioned that this issue was addressed at an international conference, which was focused at reducing maternal and perinatal-infant morbidity and mortality. 

**Suggested revisions:**

-  Although, the authors describe and analyze these data at great length, the manuscript is conspicuous by the absence of similar data about the expectant mothers from the same group. If they do not have such information or have not collected the same, it would be advisable to include historical data of this population or similar population, as collected by them or others in the literature.
- The authors have also reported that the neonatal and infant mortality was higher in the area, where the study was conducted, compared to the rest of Sri Lanka. Does this information address this disparity?
- It is not clear whether the fathers were aware of, or given information as to how this knowledge and these health issues would affect outcomes of these pregnancies, health of other members of their families and future health of their children - the authors should specify this.
- Do authors have any knowledge about the number or percentage of patients that attended these clinics in their pregnancies?
- In limitation of this study authors have addressed the issue of fathers who attend the clinic and those who do not. For those who do not, is it because they have limited resources and less knowledge?
- The authors have addressed three important issues: anemia, diabetes and eclampsia. How do they propose to use this information and implement changes if necessary, to educate the population by various measures including social awareness, impact on society, country and thus improve maternal and child health? They indeed mention that the Sri Lankan maternal health program study should change their strategy in order to improve knowledge about these conditions - this strategy should be a part of multi- prong approach.

I have read this submission. I believe that I have an appropriate level of expertise to confirm that it is of an acceptable scientific standard.
